# Unveiling microbial guilds and symbiotic relationships in Antarctic sponge microbiomes

**DOI:** 10.1038/s41598-024-56480-w

**Published:** 2024-03-16

**Authors:** Mario Moreno-Pino, Maria F. Manrique-de-la-Cuba, Marileyxis López-Rodríguez, Génesis Parada-Pozo, Susana Rodríguez-Marconi, Catherine Gérikas Ribeiro, Patricio Flores-Herrera, Mariela Guajardo, Nicole Trefault

**Affiliations:** 1 GEMA Center for Genomics, Ecology & Environment, Universidad Mayor, 8580745 Santiago, Chile; 2 Departamento de Genética Molecular y Microbiología, Facultad de Ciencias Biológicas, Pontificia Universidad Católica de Chile, Santiago, Chile; 3 Millenium Nucleus in Marine Agronomy of Seaweed Holobionts (MASH), Puerto Montt, Chile; 4 FONDAP Center IDEAL- Dynamics of High Latitude Marine Ecosystem, Valdivia, Chile

**Keywords:** Microbiome, Antarctic sponges, Sponge holobiont, Metagenome-assembled genomes, Antarctica, Extreme environment, Microbial ecology, Environmental microbiology

## Abstract

Marine sponges host diverse microbial communities. Although we know many of its ecological patterns, a deeper understanding of the polar sponge holobiont is still needed. We combine high-throughput sequencing of ribosomal genes, including the largest taxonomic repertoire of Antarctic sponge species analyzed to date, functional metagenomics, and metagenome-assembled genomes (MAGs). Our findings show that sponges harbor more exclusive bacterial and archaeal communities than seawater, while microbial eukaryotes are mostly shared. Furthermore, bacteria in Antarctic sponge holobionts establish more cooperative interactions than in sponge holobionts from other environments. The bacterial classes that established more positive relations were Bacteroidia, Gamma- and Alphaproteobacteria. Antarctic sponge microbiomes contain microbial guilds that encompass ammonia-oxidizing archaea, ammonia-oxidizing bacteria, nitrite-oxidizing bacteria, and sulfur-oxidizing bacteria. The retrieved MAGs showed a high level of novelty and streamlining signals and belong to the most abundant members of the main microbial guilds in the Antarctic sponge holobiont. Moreover, the genomes of these symbiotic bacteria contain highly abundant functions related to their adaptation to the cold environment, vitamin production, and symbiotic lifestyle, helping the holobiont survive in this extreme environment.

## Introduction

The sponge microbiome is a complex and diverse microbial community that includes mutualistic, commensalistic, or parasitic symbionts, accounting for up to 38% of the total sponge biomass^1^. The sponge and its microbial assemblage -the sponge holobiont- represent an ecological unit in which microorganisms are fundamental for the host health, nutrition, defense against pathogens, removal of byproducts, and synthesis of a broad spectrum of bioactive compounds^2,3^. These holobionts represent the primitive gut as sponges correspond to basal organisms in metazoan phylogeny, emerging as a symbiotic model to understand the foundations of complex interactions between microorganisms and their animal host^4^. High throughput sequencing of ribosomal genes revealed that sponges from temperate and tropical environments host an incredibly high number of bacterial phyla, which exceeds the diversity of phyla found in seawater, suggesting that sponges are a source of unique microbial diversity^5,6^. Pseudomonadota, Chloroflexota and Cyanobacteriota dominate sponge-associated bacterial communities, while Archaea are mainly dominated by Crenarchaeota^4,5^. Moreover, comparisons between sponge and seawater (SW) showed that fungi (e.g., Ascomycota) are mostly incidental in sponges, not supporting a sponge-specific fungal community^7–9^.

Sponges are key ecosystem engineers, providing habitat, refuge, and nutrition for many marine organisms^10^. In Antarctica, sponges can occupy up to 80% of the available substrate^11,12^. Moreover, the level of endemism within Antarctic sponges is high, reaching 68% among members of the class Hexactinellida^13^. It is still unclear how the sponge holobiont can sustain vital processes under the extreme conditions of Antarctica. Bacteria, Archaea, and Eukarya members from Antarctic sponge microbiomes show different diversity patterns with specific taxonomic signatures for this environment, the most important being the absence of cyanobacteria^10,14, 15^. Furthermore, bacterial strains isolated from Antarctic sponges reveal specific mechanisms of symbiosis and environmental adaptations^7,16^. Core functions of the Antarctic sponge microbiomes include secondary metabolite production and adaptations to a symbiotic lifestyle, like diverse bacterial defense systems^7,16^. Among these systems, the clustered regularly interspaced short palindromic repeats (CRISPR), eukaryotic-like proteins, transposases, toxin-antitoxin (T-A), and restriction-modification (R-M) systems are typically detected^7,16^. Functions related to nutrient cycling include sulfur and sulfite oxidation, ammonia and nitrite oxidation, denitrification, and polymerization and depolymerization of polyphosphates (polyP). In addition, a broad repertoire of carbon fixation pathways was identified in *Myxilla* sp. and *Leucetta antarctica*, two common Antarctic sponge holobionts, with an important contribution of light-independent pathways mediated by chemoautotrophic microorganisms^7^. However, despite these advances, many aspects of the sponge microbiome ecology are not fully understood.

Here, we used high-throughput sequencing approaches to unveil ecological patterns, community composition, and metagenome-assembled genomes (MAGs) to deepen our knowledge of the functional traits of the Antarctic sponge holobiont. By using a high number of sponge species and a combination of gene- and genome-centered metagenomic approaches, we comprehensively (i) characterize the community composition of the sponge-associated microorganisms in this extreme environment (i.e., which microorganisms are present, in what numbers or proportions, and how they relate to each other); (ii) determined the main microbial guilds in the Antarctic sponge holobiont (i.e., functional group organization that reflects the combination of microorganisms based on functional responses to environmental variables or effects on ecological processes, regardless of their taxonomic affiliation); and (iii) identified specific genomic features and functional traits of the more abundant microbial members in the Antarctic sponge holobiont (i.e., functional genes that confer environmental and symbiotic adaptation). Our findings provide further evidence to understand how the microbial symbionts could help the sponge holobiont to survive in this extreme environment.

## Results and discussion

### Distributions of bacteria, archaea, and eukaryotes associated with Antarctic sponges

Using a three-domain perspective (Bacteria, Archaea, and Eukarya), we assessed the diversity and community composition of Antarctic sponge microbiomes. The sponge species analyzed represent two classes (Calcarea and Demospongiae); six orders; 13 families, and 17 genera. To our knowledge, this is the most extensive taxonomic repertoire of Antarctic sponges analyzed to date. From the 18 sponge species analyzed by massive sequencing of the 16S and 18S rRNA genes (Supplementary Table S1), we recovered 7,417 bacterial, 40 archaeal, and 402 microbial eukaryote ASVs (Supplementary Table S2).

Bacteria/archaea diversity indices were significantly lower in the Antarctic sponge microbiomes than in the SW, without significant differences in richness. However, no statistical differences in diversity indices were observed for the microbial eukaryote communities except for the richness index (Supplementary Fig. S1 and Supplementary Table S2). The composition of the bacterial community across the sponge species was consistent with our prior studies^7,14^. Gamma- and Alphaproteobacteria dominated the sponge microbiomes, followed by Bacteroidia, Verrucomicrobiae, Planctomycetes, and Nitrososphaeria (Supplementary Fig. S2A). The main representatives of Gammaproteobacteria included Pseudomonadales, Salinisphaerales, Burkholderiales, and the UBA10353 marine group (Supplementary Fig. S3A). The main representatives of the Alphaproteobacteria were Rhodobacterales, Rhizobiales, Caulobacterales, and Rhodospirillales (Supplementary Fig. S3B). This profile, together with the lower bacteria/archaea diversity within sponges relative to SW community, and the low abundance of taxa generally used to classify sponges as high microbial abundance (HMA), i.e., Chloroflexi, Poribacteria, Actinobacteriota, Acidobacteriota, and Gemmatimonadota^17,18^, suggests that all the Antarctic sponge species analyzed here belong to the low microbial abundance (LMA) group.

Microbial eukaryotes stood out by the presence of diverse and low abundant members (Supplementary Fig. S2B). Ochrophyta and Dinoflagellata were the most dominant divisions in Antarctic sponges and SW samples, accounting for a relative abundance of 43 ± 17% and 15 ± 6%, for Ochrophyta and Dinoflagellata respectively. Within the Ochrophyta division, the Thalassiosirales order was the most represented (Supplementary Fig. S3C), while in dinoflagellates, the most abundant order was Peridiniales (Supplementary Fig. S3D). Associations with these eukaryotic members have been previously identified^14,19, 20^. Dinoflagellates, including members of the family Symbiodiniaceae, establish symbiotic relationships with various invertebrates, including sponges^21,22^. These photosynthetic dinoflagellates play a role in carbon cycling and can increase sponge bioerosion activity^23^. In contrast, diatoms are a food source for filter-feeding organisms and have been associated with degenerative processes in sponge tissue^24^.

**Figure 1:**
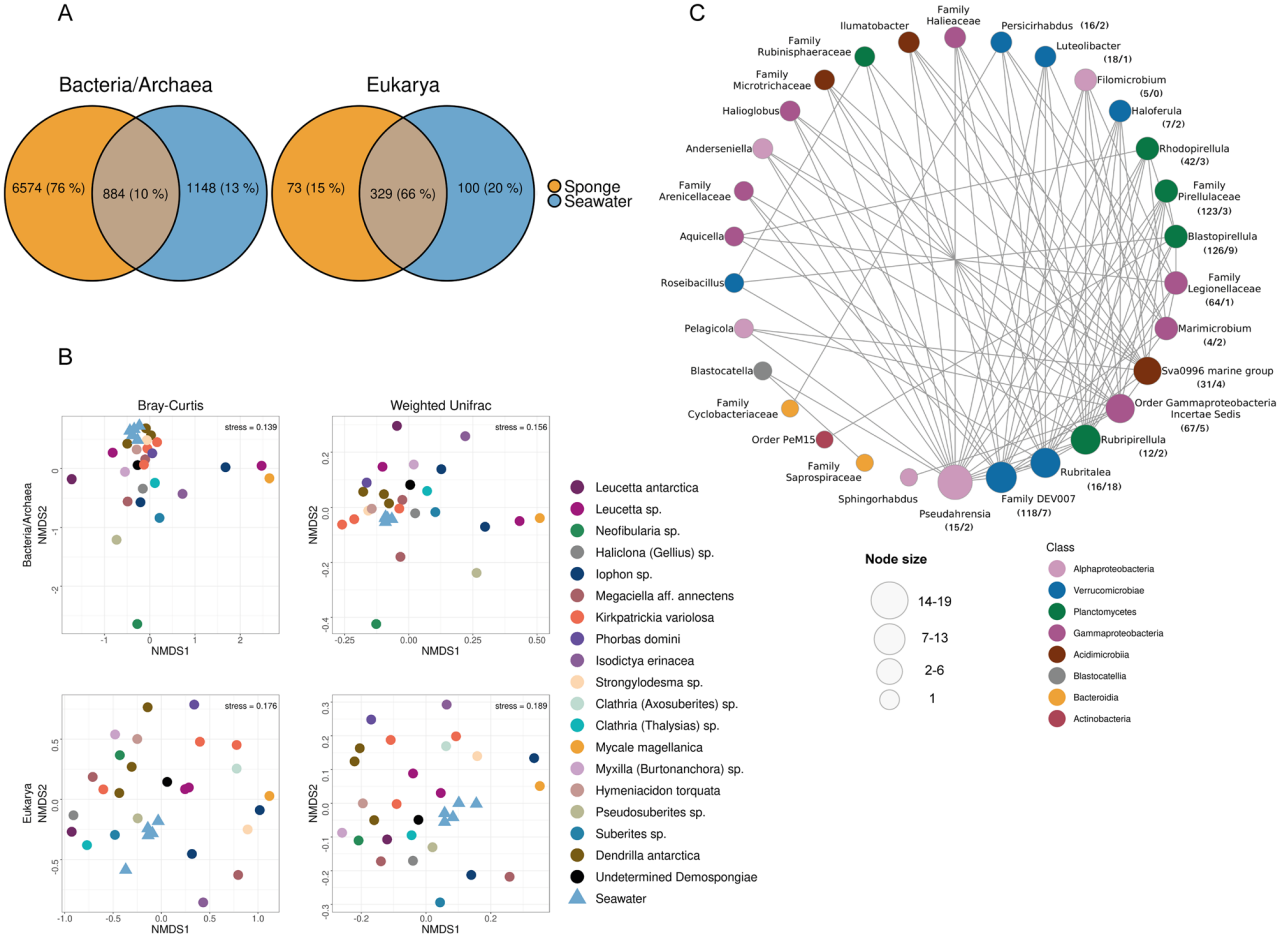
Distributions of microorganisms associated with Antarctic sponges and surrounding seawater and co-occurrence network among sponge-associated bacteria. (**A**) Shared and exclusive ASVs from the microbial communities of sponges and seawater. Exclusive ASVs were defined as those present in at least one sponge sample but absent in the SW samples, while shared ASVs were defined as those in at least one sponge sample and the SW dataset. (**B**) Non-metric multidimensional scaling (NMDS) based on Bray–Curtis dissimilarity distance (left panel) and Weighted Unifrac phylogenetic distance (right panel) for bacteria/rchaea (upper panel) and Eukarya (lower panel). Asterisks show significant differences based on the Wilcoxon test. (**C**) Co-occurrence network based on positive correlations in absolute abundance of bacteria at the genus level. Node colors indicate the taxonomic assignments at the class level. Node size is equivalent to the node degree (number of connections of each node). The values within parentheses next to each node indicate the number of ASVs exclusive to sponges and the number of ASVs shared with the seawater, respectively, for the top fifteen most abundant ASVs.

Antarctic sponges harbor a significant proportion (76%) of exclusive bacterial/archaeal ASVs, while only 10% ASVs were shared with surrounding SW microbial communities. In contrast, only 15% of the microbial eukaryote ASVs were exclusively found in the sponges, and 66% were shared between sponges and SW (Fig. 1A). These results show the high specificity of bacterial/archaeal members with Antarctic sponges and agree with previous research conducted on Antarctic sponge species^14,25^. In the case of eukaryotic communities, our previous report on eight Antarctic sponge species revealed less overlap of eukaryotes^14^. The observed variation in the quantity of sponge-specific eukaryotes can be explained by using a wider array of sponge species in the current study. The variability among sponges can be observed in the compositional (Bray–Curtis) and phylogenetic (Weighted Unifrac) dissimilarities, which do not reveal apparent clustering between the sponges for bacteria/archaea and microbial eukaryotes (Fig. 1B).

### Main co-occurrences in the Antarctic sponge microbiota

To investigate the interactions among microorganisms associated with sponges, we constructed microbial co-occurrence networks (Fig. 1C). This analysis resulted in 50 bacterial ASVs (nodes) and 115 statistically significant positive interactions between them (network edges), (Supplementary Table S3). We do not retrieve any statistically significant negative interactions for Bacteria/Archaea nor for Eukarya (negative or positive). The most highly connected cluster comprised 30 bacterial genera belonging to eight co-occurring classes, including Gammaproteobacteria, Verrucomicrobiae, Alphaproteobacteria, Planctomycetes, Acidimicrobiia, Bacteroidia, Blastocatellia, and Actinobacteria. The co-occurrence network for eukaryotes contained very few nodes, primarily represented by Ochrophyta, Chlorophyta, and Fungi (Supplementary Table S3).

The highest connectivity between the sponge associated bacteria was represented by Pseudahrensia (Pseudomonadota) (Fig. 1C). Considering that interactions among microbial communities within holobionts can influence the dynamics and stability of symbiosis^26^, the absolute predominance of positive interactions among bacteria in the microbiome of Antarctic sponges is noteworthy. This result contrasts with previous findings from the global survey of sponge microbiomes, which indicated a higher likelihood of negative interactions among operational taxonomic units (OTUs) within the core microbiome^5^, however it does not include Antarctic or cold-water sponges. The extensive presence of positive links in the Antarctic sponge microbiome suggests that bacterial members establish more cooperative interactions than in other environments, ultimately benefiting the sponge holobiont in a challenging environment with seasonally limited nutrients and energy.

### Microbial guilds and symbiosis features in the Antarctic sponge holobiont

Whole-metagenome shotgun sequencing was performed on a subset of eight sponge species from the original Antarctic sponge microbiomes analyzed. These eight sponge species were selected because they correspond to some of the most abundant species in Antarctica. The percentage of metagenomic reads annotated for bacteria, archaea, and eukaryotes was 86 ± 10%, 4 ± 3%, and 10 ± 8%, respectively.

**Figure 2:**
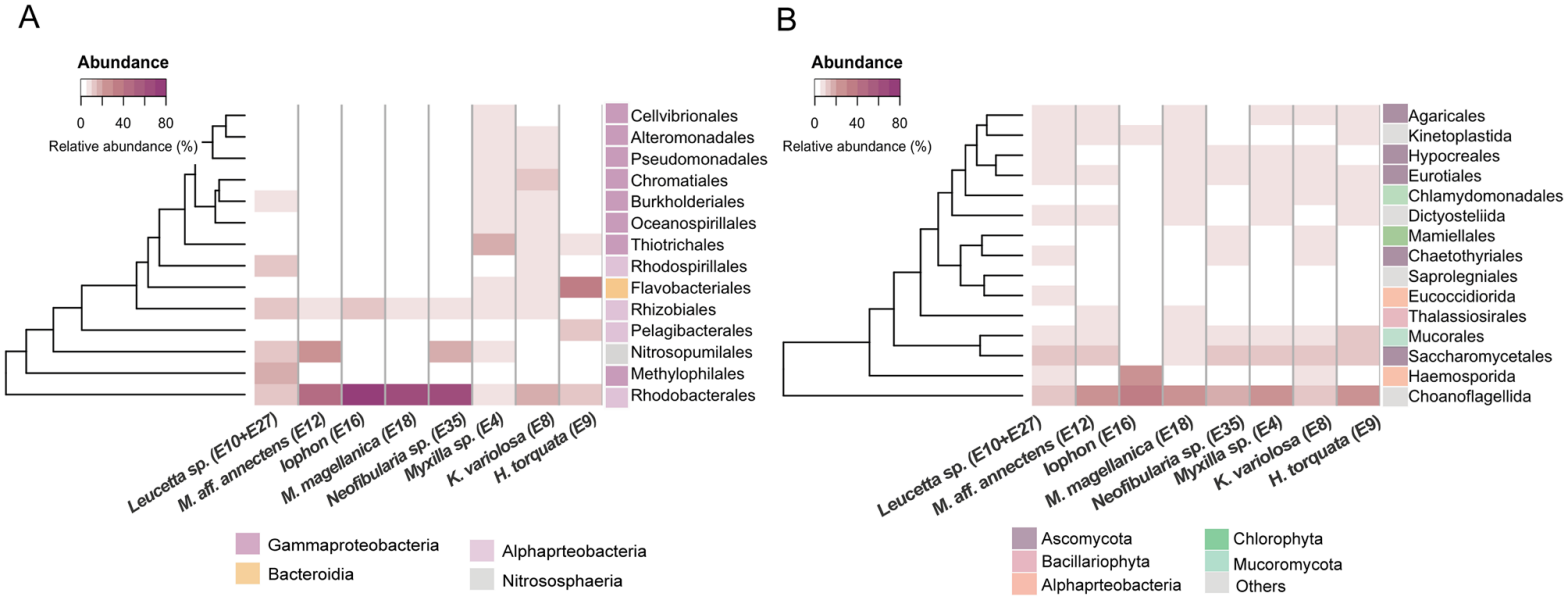
Microbial community composition based on the functional annotation of metagenomic reads of Antarctic sponges. Taxonomic classification was conducted at the order level using the number of reads corresponding to the metagenome with the lowest number (15,475,241 reads, *Myxilla* sp. (E4), without considering E9 (*Hymeniacidon torquata*) and E16 (*Iophon* sp.)), where all reads were used. Taxonomic annotation was done using Kaiju and the nr-euk NCBI database.

Overall, the taxonomic profile of metagenomic data assigned to bacteria and 16S amplicons were more similar than the taxonomic profile of metagenomic reads assigned to eukarya and 18S amplicons, however, in both comparisons exist discrepancies (Fig. 2; Supplementary Fig. S2A; and Supplementary Table S4 and S5). For example, Pseudomonadales, the top 3 order within Bacteria/Archaea, appears in the 16S rRNA gene amplicons with much higher relative abundance than in the metagenomic dataset. On the other hand, Salinisphaerales and Caulobacterales only appear in the 16S dataset. In the case of the annotated reads for eukaryotes, a significant proportion corresponded to fungal sequences (44 ± 4%), with Saccharomycetes, Aconoidasida, and Mucoromycota dominating only in the metagenomic datasets (Fig. 2, Supplementary Fig. S2B). These discrepancies could be attributed to differences primarily related to the databases used for taxonomic annotation of the 16S rRNA gene and metagenomes, as well as the 18S rRNA gene primer set used, which may provide less taxonomic coverage for fungi^27^.

For bacteria, metagenomic reads were mainly classified according to eggNOG in the 'extracellular structures' category (15 ± 1%), followed by 'amino acid metabolism and transport' (10 ± 1%), and 'replication and repair' (10 ± 3%) categories. Other categories accounted for variable proportions, ranging between 8 and 0.003% (Supplementary Table S6).

In the case of the reads assigned to eukaryotes, most annotated sequences are in the categories of 'post-translational modification, protein turnover, and chaperones' (11 ± 3%), 'unknown' (11 ± 3%), and 'energy production and conversion' (10 ± 12%). Notably, approximately 45 ± 4% of these eukaryotic reads were identified as fungi and functionally classified as ‘unknown functions’ (12 ± 5%), followed by ‘replication, recombination, and repair’ (12 ± 4%); and ‘post-translational modification, protein turnover, and chaperones’ (11 ± 1%) (Supplementary Table S6).

**Figure 3:**
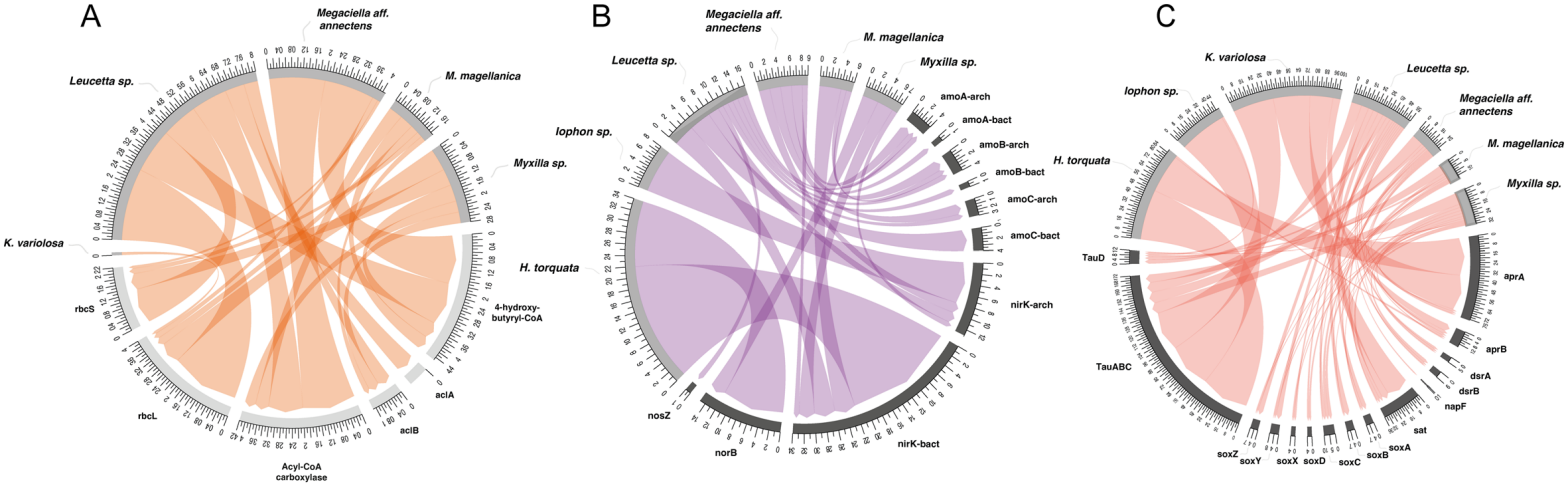
Metabolic potential of the main microbial guilds detected in the Antarctic sponge metagenomes. The Circos plot illustrates the abundance of genes associated with carbon (**A**), nitrogen (**B**), and sulfur (**C**) pathways. The abundance was calculated based on gene abundance using RPKG (reads per kilobase per genome equivalent) and normalized by the average genome size of the genes involved in each pathway.

Bacterial, archaeal, and eukaryotes assigned reads allow us to identify microbial guilds involved in carbon fixation and nitrogen and sulfur cycling in the Antarctic sponge microbiomes (Fig. 3 and Supplementary Table S7). For carbon fixation, the key genes for the Calvin-Benson-Bassham (CBB) cycle- *rbc*L and *rbc*S- were present in all sponges, with a high enrichment in *Leucetta* sp. (Fig. 3A)*.* These genes were almost exclusively found in the metagenomic reads from the microbial eukaryotes (Supplementary Fig. S4). The key marker genes for the thaumarchaeal 3-hydroxypropionate/4-hydroxybutyrate (HP/HB) cycle (4-hydroxy-butyryl-CoA and Acyl-CoA carboxylase) and for the reductive tricarboxylic acid rTCA (*acl*A and *acl*B) were mainly detected in *Leucetta*, *M. aff annectens*, *M. magellanica*, and *Neofibilaria* sp. (Fig. 3A)*.* The microbiome diversity from sponges distributed between South America and Antarctica indicates that they differ in terms of the autotrophic fraction, with South America sponges having a higher abundance of photosynthetic microorganisms while sponges from Antarctica, the highest abundance of chemosynthetic^28^. Considering that Antarctic sponges harbor an important proportion of diatoms and dinoflagellates, which exclusively perform carbon fixation via photosynthesis, we suggest that these microbial eukaryotes could significantly contribute to the overall carbon cycle in Antarctic sponges. Furthermore, due to the light regimen in Antarctica, dark CO_2_ fixation could support the C pool in these sponge holobionts during the winter season^7^.

For the nitrogen cycle, ammonia, nitrate, and nitrite oxidation genes were present in all the sponge metagenomes (Fig. 3B, Supplementary Fig. S4, and Supplementary Table S7). Functional equivalence in ammonia and denitrification processes is a common occurrence across tropical, temperate, and Mediterranean sponges, where phylogenetically distant sponges host microorganisms that tend to carry similar functions^26,27^. Furthermore, we found coding genes for taurine degradation, dissimilatory sulfite reduction, and thiosulfate oxidation (Fig. 3C). Genes related to the symbiotic lifestyle and cold adaptations (i.e., cold shock proteins, osmotic stress, protein folding, and oxidative stress) were also highly abundant in all metagenomes (Supplementary Table S8).

### Genome-centered metagenomic approach in Antarctic sponge holobionts

**Figure 4:**
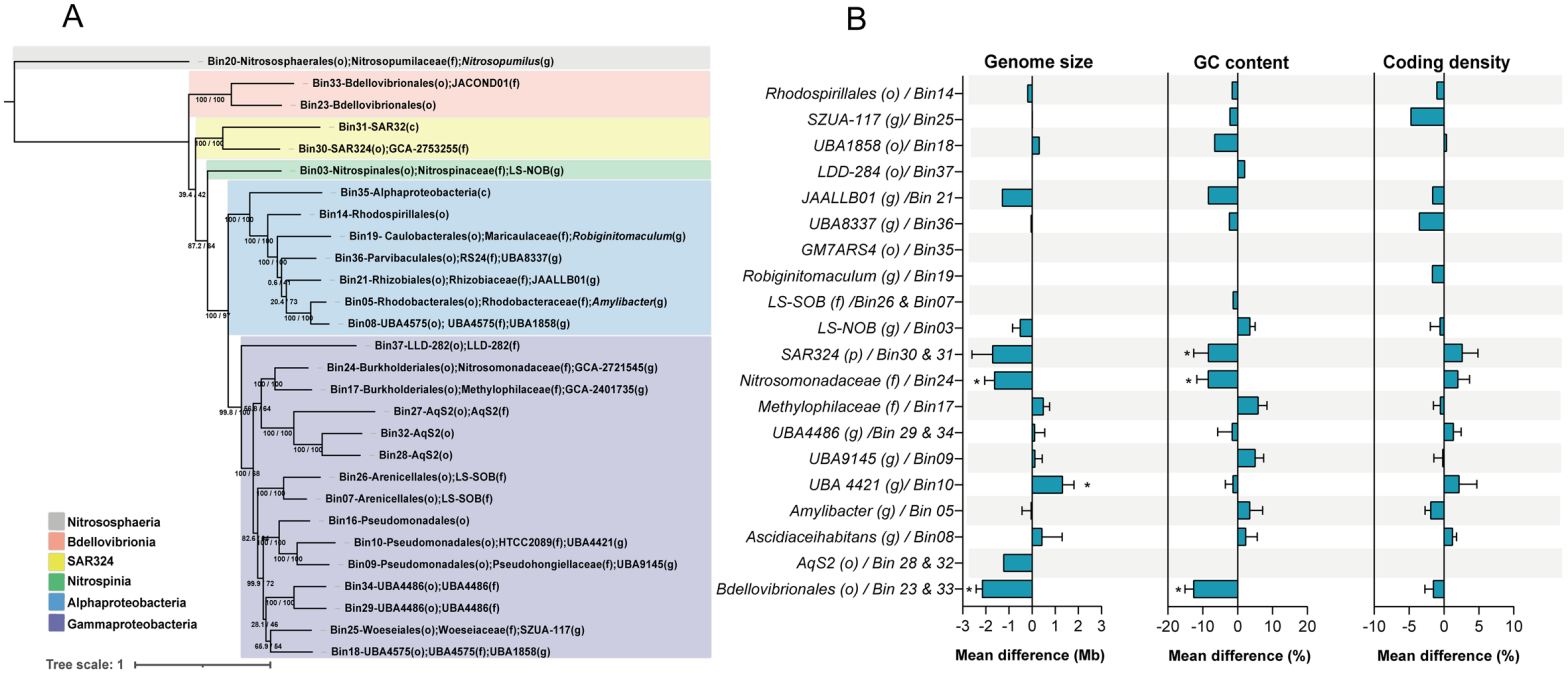
Phylogenomic tree representing the taxonomic affiliation of bacterial and archaeal MAGs and genomic features in associated vs. free-living bacteria. In (**A**), the phylogenomic tree is based on 25 single-copy genes. The color denotes the classification of MAGs at the class level. The ultrafast bootstrap (UFboot) and the Shimodaira–Hasegawa approximate likelihood-ratio test (SH-aLRT) consensus tree were inferred from 1,000 replicates. The SH-aLRT and UFBoot are given as branch support values. The taxonomic assignment at the deepest level is shown for each bin in parentheses: p = phylum, o = order, f = family, and g = genus. In (**B**), each graph shows the mean difference and standard deviation of genome size in Mb, GC content, and coding density between genomes reconstructed from associated and free-living bacteria with at least 75% completeness. Significant differences in genome size (p-value < 0.001) and GC content (p-value < 0.012) are indicated with an asterisk.

To gain further insight into the identity and symbiotic features of the bacterial guilds representatives, we used a genome-centered metagenomic approach. After re-replication, we obtained 28 high- and medium-quality Metagenome-Assembled Genomes (MAGs), with a median of 89 ± 6% completeness, 0.8 ± 0.7% contamination, and 156 ± 103 contigs. They belong to Gammaproteobacteria (15 MAGs), Alphaproteobacteria (7 MAGs), Bdellovibrionia (2 MAGs), Deltaproteobacteria SAR324 (2 MAGs), Nitrospinia (1 MAG), and Nitrososphaeria (1 MAG) (Fig. 4A). Their taxonomic classification indicates that they correspond to the most abundant bacterial members in each guild (Supplementary Table S9), agreeing with our tag sequencing and metagenomic results (see Supplementary Fig. S3 and Fig. 2). Notably, whole-genome comparisons indicated that these MAGs shared only between 76 and 95% of Average Nucleotide Identity (ANI) with the genomes available in the Genome Taxonomy Database (GTDB), showing a high novelty in the genomes of microbial symbionts of Antarctic sponges (Supplementary Table S9).

In the Antarctic environment, due to its poor sampling and knowledge of its microbial diversity, it is common to detect previously undescribed bacteria that represent entire taxonomic groups without parallels in public databases. On the other hand, the host evolutionary history plays a significant role in structuring the community of symbionts^5^. The long history of isolation and, therefore, the high marine endemicity of Antarctica^29^ could partially explain the high diversity and novelty of the genomes assembled from sponges in this environment. However, the low representation of genomic data from the Antarctic marine environment^30^ makes it difficult to interpret these results further, highlighting the urgency to increase the coverage of microbial symbionts from extreme environments in genetic databases for a more comprehensive understanding of the novelty of some microbial groups from Antarctic sponges.

Through the comparison of genomic-wide signatures of microbial symbiosis, i.e., genome reduction and GC- content of close members to our MAGs, we found evidence of genome streamlining in several clades of Antarctic sponge symbionts. Specifically, we found significant differences in the genome size and GC content of host-associated and free-living members of Bdellovibrionales and Nitrosomonadaceae. The genome size was extremely reduced in Bin23 and Bin33 (Bdellovibrionales) (1.4. ± 0.2 Mbp) and Bin24 (Nitrosomonadaceae) (1.34 ± 0.1 Mbp) in comparison to their free-living counterparts (3.4 ± 0.6 Mbp). Similarly, the GC-content in these sponge symbiotic bacteria was lower than in their free-living counterparts. Bdellovibrionales Bin23 and Bin33 had a 38.8% ± 0.6%, and Nitrosomonadaceae Bin24 had a 39.9% ± 0.4% compared to 48.3% ± 4.5% GC in free-living bacteria. This reduction in genome size and GC content was accompanied by an increase in coding density (Fig. 4B).

However, in the case of the Gammaproteobacteria marine group UBA44421, we observed significantly larger genome sizes and slightly lower GC content- although not statistically significant- in the genomes of free-living representatives of these taxa (Fig. 4). This suggests that members of UBA44421 are more likely to be free-living or facultative symbionts, rather than obligate sponge symbionts. Although it is known that obligate symbionts can undergo gene loss for metabolic processes provided by the host through Horizontal Gene Transfer (HGT), this mechanism can also facilitate functional gains and genome expansion, as observed in microorganisms associated with sponges^32–34^, corals^35^, insects^36^, algae and plants^31,37^.

**Figure 5:**
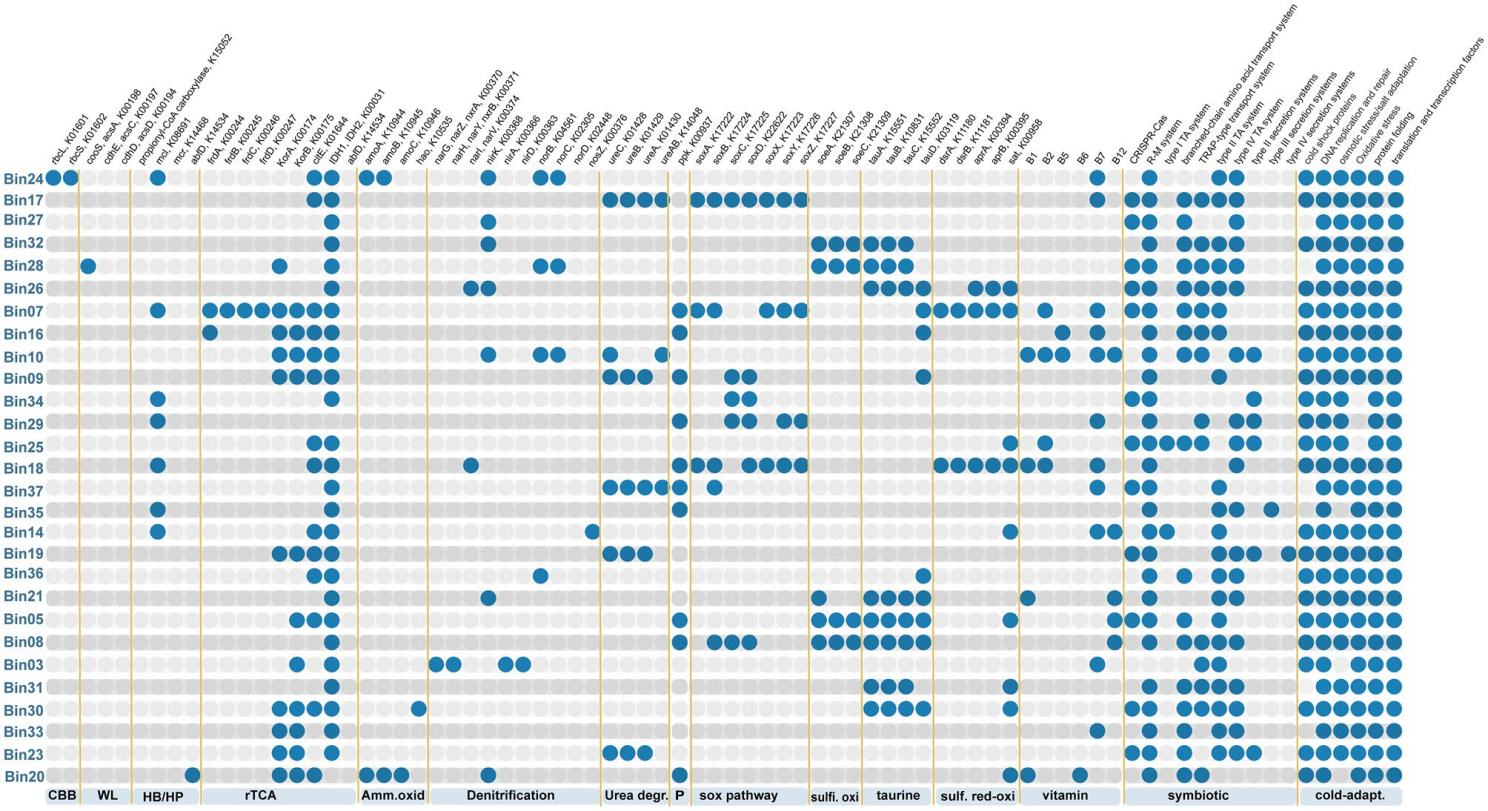
Metabolic potential predicted for MAGs obtained from Antarctic sponges. Bubbles show the presence or absence of genes related to carbon, nitrogen, sulfur, phosphorus, vitamin production, and symbiotic and cold adaptation. CBB = Calvin–Benson–Bassham cycle. WL = Wood–Ljungdahl pathway. rTCA = Reductive tricarboxylic acid cycle. Amm.oxid = Ammonia oxidation. Urea degr. = urea degradation. P = polyphosphate degradation. Sulfi.oxi = Sulfite oxidation. sulf.red-oxi = sulfur reduction–oxidation. cold-adapt. = cold-adaptation.

We identified the genes related to carbon and nitrogen metabolisms in all the reconstructed MAGs (Fig. 5). In Nitrosopumilus Bin20 (ammonia-oxidizing archaea- AOA), we detected genes responsible for autotrophic carbon fixation utilizing the HP/HB cycle and ammonia oxidation and nitrite oxidation and reduction. In the case of Nitrosomonadaceae Bin24 (ammonia-oxidizing bacteria- AOB), genes related to the CBB cycle, ammonia oxidation, and nitric oxide reduction were detected. Bin03 (nitrite-oxidation bacteria-NOB) also exhibited genes encoding nitrite oxidoreductase (Fig. 5). Nitrosopumilus Bin20 was primarily present in *Megaciella* aff. *annectens* (13%), with less representation in *Neofibularia* (5%), *H. torquata* (4%), and *K*. *variolosa* (2%). On the other hand, Nitrosomonadaceae Bin24 was mainly associated with *Megaciella aff. annectens* (12% relative abundance). Meanwhile, the NOB Bin03 was found in high abundance only in *Myxilla* sp. (65%) while showing less than 1% relative abundance in the other sponge species (Supplementary Fig. S5). Although there was no significant difference in the relative abundance of AOA Bin20 (3.14 ± 4.4%) and AOB Bin24 (1.99 ± 4.1%) among the sponge microbiomes, we observed that Bin20 outnumbered Bin24 in specific metagenomes. Bin20 was 39, 32, 2.4, and 2.3-fold more abundant than Bin24 in *K. variolosa, H. torquata, Neofibularia,* and *Leucetta* sp.*,* respectively (Supplementary Table S9).

A higher abundance of AOA over AOB was also reported in cold-water and deep-sea sponges from the northern hemisphere^38,39^. AOA are well-adapted to ammonia-limited conditions, whereas AOB dominates under high ammonia inputs^40,41^. Thus, ammonia produced as a metabolic waste by sponges can lead to competition between AOA and AOB for metabolization^38^. Nitrification, the process of oxidation of ammonia to nitrate, is primarily carried out by nitrite-oxidizing bacteria (NOB), which often occurs close to AOA to convert ammonia directly to nitrate^42^. Our study indicates the enrichment of AOA and NOB in several of the analyzed Antarctic sponges, suggesting the potential for complete nitrification processes within these microbiomes. However, the low abundance of NOB in most Antarctic sponge species studied here may lead to incomplete nitrite oxidation. This incomplete nitrification could be attributed to different nutrient fluxes and nitrite release, as observed in deep-sea sponges^43^.

In the sulfur-oxidizing bacteria (SOB) Bin07, we found the key genes encoding the reductive tricarboxylic acid (rTCA) cycle and sulfur reduction (*dsr, apr,* and *sat* genes) (Fig. 5). However, sulfur cycling, similar to carbon and nitrogen, was widespread among the binned genomes. Sulfite oxidation using membrane-bound sulfite dehydrogenase (*soeABC* genes) and taurine degradation- leading to sulfite production- was found in the Alphaproteobacteria Bin05, Bin08 and Bin21, Gammaproteobacteria Bin30, SAR324 Bin30 and the AqS2 Bin28 and Bin32. It has been proposed that organosulfur compounds derived from the host play a crucial role within the sponge holobiont, as microorganisms can use them as energy and organic sources (Engelberts et al., 2020). Taurine dioxygenase, the enzyme responsible for converting taurine to sulfite, has been previously reported in sponge microbiomes from the Northeast Atlantic, mostly affiliated to Acidobacteriota, Actinobacteriota, and Alpha- and Gammaproteobacteria^44,45^. Taurine is also a key intermediate for host-symbiont interaction, as noted in the tropical sponge *Ianthella basta*^46^. In this sponge, the dominant gammaproteobacterial symbiont, ‘*Candidatus* Taurinisymbion ianthellae’, uses taurine-derived carbon and nitrogen and oxidizes the dissimilated sulfite into sulfate for export to the host.

We also detected genes related to symbiosis in all MAGs (Fig. 5 and Supplementary Table S9). SOB Bin07 and AqS2 Bin28 were particularly enriched in CRISPR-Cas, R-M, and branched-chain amino acid transport systems. These functions are commonly found in the sponge symbiont genomes and are essential for establishing a symbiotic relationship^47,48^. Moreover, these symbionts can use amino acids as an energy source^48^, further emphasizing their role in the sponge-microbe relationship. Furthermore, we found the complete pathways for essential vitamin biosynthesis in all MAGs including thiamine (B1), riboflavin (B2), pantothenate (B5), pyridoxine (B6), biotin (B7), cobalamin (B12) (Supplementary Table S10).

Another important functions detected in the MAGs for the symbiotic lifestyle were carbohydrate-active enzymes (CAZys). CAZys are families of structurally-related catalytic and carbohydrate-binding modules (or functional domains) of enzymes that degrade, modify, or create glycosidic bonds. These enzymes may play critical roles in symbiosis facilitating adherence to host tissue^49,50^ In lichen symbionts, CAZys have shown to enhance phototrophic CO2 assimilation using external carbon sources^51^. The CAZys in the MAGS were mainly represented by several glycoside hydrolases (GH) and glycosyltransferases (GT), which are involved in hydrolysis, transglycosylation, and biosynthesis of glycosidic bonds, followed by lesser abundant enzymes such as Auxiliary activities (AAs) (Supplementary Fig. S6 and Supplementary Table S10). The GH and GT families are relevant in symbiosis as they facilitate symbiont adherence to the sponge tissue (e.g., bacterial cellulose synthase and GHe)^52,53^. Moreover, GH are involved in chitin degradation and peptidoglycan synthesis^53^. Chitin is a sponge structural polysaccharide and it seems to have a role in establishing symbiotic associations in the demosponges^54,55^.

The presence of degradative enzymes in the genome of Antarctic sponge symbionts suggests their contribution to the breakdown of chitin, potentially supporting the establishment and persistence in the sponge tissue. In the case of host-associated bacteria, GT family enzymes involved in lipopolysaccharide (LPS) biosynthesis play a role in overcoming the sponge immune system, aiding in the discrimination between beneficial symbionts and potential pathogens^56^.

Finally, all the analyzed bins show evidence of cold adaptation, indicating these functions constitute a fundamental aspect of symbionts within Antarctic sponges (Fig. 5 and Supplementary Table S10). These elements include cold shock proteins, DNA replication, and repair mechanisms, osmotic stress responses, salt adaptation, oxidative stress responses, protein folding, and translation and transcription factors. These features, typically seen in psychrophilic microorganisms^57–59^, are enriched in Antarctic sponge symbionts^16^. Given the well-documented environmental adaptation of the sponge microbiome to local conditions (e.g., cold seep, deep-sea, shallow sponges), we hypothesize that cold adaptations might play a crucial feature in maintaining the functionality of symbiont metabolisms and overall health of the Antarctic sponge holobiont.

## Conclusions

The extremely harsh conditions in Antarctica provide an excellent scenario to study the diversity and functional interaction within the sponge holobiont and how microbiome adaptation has influenced its relationship with its environment. Our study uncovers that Antarctic sponges harbor microbial symbionts that are crucial for maintaining the health of the sponge holobiont. These symbionts are specialized in synthesizing and providing essential nutrients to the sponge while also being adapted to thrive in cold environments. Particularly, the Western Antarctic Peninsula is experiencing accentuated changes in its climate pattern with a continued warming trend and consequently increased vulnerability to sea-ice loss^60^. These changes impact the benthic communities, the coupling between benthic and pelagic ecosystems, and primary production^61^.  Moreover, the recruitment and growth of sponges in Antarctica are closely linked to sea-ice dynamics^61,62^, and some Antarctic sponges have shown a limited range of tolerance to increased temperatures^63^. However, in other climate change scenarios, sponges appear more resilient than other dominant benthic organisms, like corals, as they are less affected by ocean warming or acidification^64^. All these factors highlight the pivotal role of the Antarctic sponge holobiont in understanding the symbiotic relationship between animals and microbes in extreme environments. Furthermore, studying this relationship can provide insights into how the polar sponge holobiont will respond to future climatic pressures.

## Methods

### Sample collection

Sponge samples (n = 26) were collected by scuba diving in January 2013, February 2014, and January 2015 from Fildes Bay, King George Island, South Shetlands, Antarctica. Sponge samples were maintained individually in plastic bags containing natural seawater (SW) until processing (within 3 h post sampling). SW samples were collected using a 5 L Niskin bottle, approximately 5 m from each sponge sampling site. Sponges were collected between 5 and 27 m in depth (Supplementary Table S1). SW samples were prefiltered on board through 150 μm pore mesh to remove large particles, stored in acid-washed carboys, and kept in the dark until processing in the lab at the scientific station ‘Profesor Julio Escudero’ from the Instituto Antártico Chileno (INACH). Salinity, fluorescence, oxygen, and temperature data were obtained using the SBE 911 plus (SeaBird) CTD profiler. The INACH issued the permissions for sampling (permits # 498/2013; 17/2014, and 03/2015).

### Sponge and seawater processing and DNA extraction

Sponge samples were rinsed with filtered SW, cleaned under a stereomicroscope, and stored at −80 °C until further processing at the laboratory in Santiago (GEMA Center, Universidad Mayor). Triplicate tissue samples of ~ 0.5 cm^3^ were extracted with a sterile scalpel blade from each sponge sample. The sponge-associated and SW microbial community processing was done according to Rodríguez-Marconi et al.^14^. Briefly, the sponge tissue was carefully disrupted, followed by filtration and serial centrifugation, and high-molecular-weight DNA extraction was carried out on the resulting pellet with the PowerSoil DNA Isolation Kit (MOBIO).

To analyze the surrounding planktonic community, SW samples were subjected to filtration using filters of different pore sizes: 20 μm (NY20), 3 μm (GSWP), and 0.2 μm (GPWP) with a diameter of 47 mm (Millipore), using a Swinnex holder system and a peristaltic pump (Cole Parmer) set at a speed of 1–600 rpm. DNA extraction was performed using 5 M NaCl and N-cetyl N, N, N trimethylammonium bromide (CTAB) extraction buffer and a conventional phenol–chloroform method.

### 16S rRNA and 18S rRNA gene amplicon sequencing and data processing

For analyses of the bacterial communities, the hypervariable region V4 of the 16S rRNA gene was amplified using F515 and R806 primer pairs^65^. For analyses of the microbial eukaryotic communities, the hypervariable region V9 of the 18S rRNA gene was amplified using 1391f. and EukBr primer pairs^66^. PCR reactions and Illumina MiSeq sequencing were performed as described in Rodríguez-Marconi et al.^14^. Primer sequences were removed using cutadapt^67^ and raw reads were demultiplexed using QIIME2 and processed on a per-run basis for Amplicon Sequence Variant (ASV) inference using DADA2 package version 1.20.0^68^ within the R environment version 4.1.3 (R Development Core Team 2013).

For the 16S rRNA gene, trimming parameters were trimLeft = c(0,20), for the 2015 samples, truncLen = c(150,150), truncQ = 2. Only sequences matching the amplicon length of 231–238 bp were maintained. For the 18S rRNA gene, reads were filtered with the following parameters: trimLeft = c(0,27), truncQ = 2, maxN = 0, maxEE = c(2,2). Forward and reverse reads were merged using the minOverlap = 15 and = 30 for the 16S and 18S rRNA genes, respectively. Chimeras were removed using removeBimeraDenovo with the consensus method. 16S taxonomy was performed using the Silva NR99 v138.1 database^69^ and 18S using the PR2 database version 4.14^70^, with a minboostrap = 80. For the 16S rRNA gene, non-assigned ASVs at the phylum level, singletons, and ASVs assigned to mitochondria and chloroplasts were removed. For the 18S rRNA gene, non-assigned ASVs at the Kingdom level, singletons, and ASVs assigned to metazoa, bacteria, and chloroplasts were removed.

Sequences from SW samples from the three size fractions were merged using the Phyloseq package version 1.36.0^71^. Richness (Chao1) and diversity (Simpson and Shannon) indexes were calculated using the Phyloseq package. The Wilcoxon test was used to compare means. For beta diversity analysis, only ASVs with a mean relative abundance > 0.01% for Bacteria/Archaea and Eukarya were kept. Hellinger transformation was applied over the filtered ASVs. Exclusive ASVs were defined as those present in at least one sponge sample but absent in the SW samples, while shared ASVs were defined as those present in at least one sponge sample and the SW dataset.

Ordination plots were constructed using Bray–Curtis dissimilarity and Weighted UniFrac phylogenetic distances with Phyloseq. For creating barplots and heatmaps, the pre-filtered ASVs of the same sponge species were merged, Hellinger-transformed, and converted to relative abundances. Then, ASVs were agglomerated at class, division, and order levels. ASVs with a relative abundance < 1% were grouped into the same label for barplot visualization.

### Co-occurrence networks inference

For rarefaction, data were subsampled to an even depth of the smallest sample using the phyloseq package. For Bacteria/Archaea, only the genera identified in at least one-third of the sponge samples were kept. The network analysis was performed using the CoNet plugin version 1.1.1.beta^72^ of Cytoscape version 3.9.1^73^. Pairwise correlations between genera were carried out by combining the Pearson and Spearman measures and requesting the network intersection. The correlation threshold for both methods was set to 0.7 (for positive and negative correlations). We avoided correlations between pairs of genera with double-zero values in more than half of the sponge samples. The P-value threshold was set to 0.05 (1,000 iterations). Simes method and Benjamini–Hochberg correction combined the networks from each measure. The resulting networks were visualized with a degree-sorted circle layout, implemented within Cytoscape. Statistical analysis and topological properties were conducted with the NetworkAnalizer plugin^74^.

### Metagenomic sequencing and analyses

Metagenomic sequencing from 9 microbiome samples (see Supplementary Table S1 for details). Adapter removal and trimming were performed using skewer v0.0.2 (parameters per default) and bbduk v38.18 (maq = 28 overwrite = true -Xmx40g)^75^, respectively, using a quality threshold of Q > 28. The pair-end reads were merged using PEAR v0.9.11(minimum overlap 20). To discard possible host contamination, sequence reads were mapped against two sponge reference genomes (*Amphimedon queenslandica*, (NC_008944.1) and *Ephydatia muelleri* (GCA_013339895.1) using Bowtie2 v2.4.4 (-reorder -very-sensitive-local -q -no-unal -S)^76^. For metagenomic analysis, the remaining reads were individually assembled per sponge species using SPAdes v3.15.3 (−k 77, 87, 97, 109, 121 -only-assembler)^77^, and analyzed according to Moreno-Pino et al.^7^. To assess the community composition of the metagenomic reads, the taxonomic annotation at the read level was performed using Kaiju v1.7.4 with the nr-euk-12-20 database (parameters per default)^78^. To compare the taxonomic composition between metagenomes, normalized reads were subsampled to an even depth, using the smallest depth sample, i.e., *Myxilla* sp., except for E16 and E9, where all reads were used. To evaluate the metabolic potential, ORFs were aligned using eggNOG mapper v.1 against the eggNOG v4.5 with parameters per default)^79^.

### Obtention of metagenome-assembled genomes (MAGs)

For MAG analysis, the non-sponge reads obtained from metagenomic analysis (see above) were co-assembled using SPAdes v3.15.3^77^ with a K-mer of 127 after evaluating different K-mer sizes, using metaQUAST. Bins were constructed using MaxBin2 v2.2.7 (-prob_threshold 0.9 -verbose -min_contig_length 1500)^80,81^ and Metabat2 v2.2.15 (-m 1500 ;2000 and 2500, with a min. contig Length of 1000)^82^. MAGs obtained in this process were curated using sequence composition and differential coverage in Anvi'o v7^83^. Dereplication was performed using DAS-tool (-write_bins 1 -write_unbinned 1 -search_engine blast) v1.1.3^84^ and MASH, ANIm, and pyANI tools^85^. Completeness and contamination of MAGs were determined with CheckM^86^. Only high- and medium-quality MAGs (> 90% of completeness, < 5% of contamination, n = 14, and > 50% of completeness, < 10% of contamination, n = 14, respectively) were kept for further analyses.

Genome annotation was performed using eggNOG mapper v.1 against the eggNOG v4.5^79^, BlastKOALA^87^, and CAZy database using dbCAN. Taxonomic classification was resolved using GTDB-tk tool v1.5.0^88^ with the GTDB r202. The phylogenomic analysis was performed following the GtoTree workflow (v1.6.11)^89^. The alignment was trimmed with trimAI (-automated1), and the phylogenomic inference was performed using iqtree2 with Shimodaira–Hasegawa approximate likelihood-ratio test (SH-aLRT) and ultrafast bootstrap approximation (UFBoot) branch test (-alrt 1000 -B 1000) and visualized in Itol v6^90^.

The comparative genomic analysis was based on the genome size, GC density, and coding gene density genomes available in GTDB r202. Thus, only available genomes from the same level of the deepest taxonomic assignment for each MAG and with at least 75% completeness were included. Genomes were categorized as host-associated or free-living. Significant differences between lifestyles were obtained using the Two-stage linear step-up procedure of Benjamini, Krieger, and Yekutieli, with Q = 5% in the Graphpad Prism software.

### Supplementary Information

Supplementary Information 1.

Supplementary Information 2.

## Data Availability

Raw amplicon sequences from 2013 were deposited in SRA under PRJNA287634 BioProject number. Raw amplicon sequences from 2014 and 2015 were deposited in SRA under PRJNA870453 and PRJNA901435 for sponge microbiome and SW, respectively. Raw metagenome sequences were deposited in SRA under PRJNA874040. MAGs were deposited under BioProject number PRJNA871246 and https://figshare.com/s/14d95f4a82f89c5c29b9.
