# Supplementary material for: Unveiling microbial guilds and symbiotic relationships in Antarctic sponge microbiomes
Source: Sci Rep. 2024 Mar 16;14:6371. doi: 10.1038/s41598-024-56480-w (PMC10944490; doi:10.1038/s41598-024-56480-w)
Supplement: Supplementary file 1 — Supplementary Information 1. [file 41598_2024_56480_MOESM1_ESM.pdf]

Supplementary Information

Unveiling Microbial Guilds and Symbiotic Relationships in Antarctic Sponge Microbiomes

Mario Moreno-Pino[a,b], Maria F. Manrique-de-la-Cuba[a], Marileyxis López-Rodríguez[a], Génesis Parada-Pozo[a,c], Susana Rodríguez-Marconi[a], Catherine Gérikas Ribeiro[a], Patricio Flores-Herrera[a], Mariela Guajardo[a], Nicole Trefault*[a,c,d].

[a]Centro GEMA – Genómica, Ecología & Medio Ambiente, Facultad de Ciencias, Universidad Mayor, Santiago, Chile.

[b]Departamento de Genética Molecular y Microbiología, Facultad de Ciencias Biológicas, Pontificia Universidad Católica de Chile, Santiago, Chile.

[c]Millenium Nucleus in Marine Agronomy of Seaweed Holobionts (MASH), Chile. [d]FONDAP Center IDEAL- Dynamics of High Latitude Marine Ecosystem, Chile.

Keywords: Microbiome, Antarctic sponges, sponge holobiont, metagenome-assembled genomes, Antarctica, extreme environment.

Running title: Microbiomes of Antarctic sponges.

Supplementary Figures

Supplementary Figure S1: Alpha diversity analysis of microbial communities associated with Antarctic sponges and surrounding seawater**.** Violin plots show Chao richness estimator, Simpson diversity, and Shannon diversity for Bacteria/Archaea (upper panel) and Eukarya (lower panel). Asterisks denote $p<0.01$ according to the Wilcoxon Mann-Whitney test.

Supplementary Figure S2: Microbial community composition of Antarctic sponge microbiomes based on 16S and 18S rRNA genes. A) Bacteria/Archaea taxonomic composition at the class level according to the Silva database. B) Eukarya taxonomic composition at the division level according to the PR2 database.

Supplementary Figure S3: Relative abundance of the dominant orders of Bacteria and Eukarya associated with Antarctic sponges. Top 20 dominant orders of Bacteria/Archaea (A) and Eukarya (B). Asterisks indicate taxonomic assignments according to the NCBI 16S rRNA database when the Silva database was unable to assign at the order level.

Supplementary Figure S4: Metabolic potential of the main members of the microbial guilds detected in the Antarctic sponge metagenomes. Circos plots represent the percentage of genes related to carbon (orange), nitrogen (purple), and sulfur (red) pathways.

Supplementary Figure S5: MAG distribution among the Antarctic sponge microbiomes. The abundance of each MAG is measured based on RPKM (reads per kilobase per million reads mapped).

Supplementary Figure S6: Number of genes related to carbohydrate-active enzymes (CAZy) modules in the genomes of the 28 MAGs. The main modules of CAZy 10 glycosyltransferases (GT) and glycoside hydrolases (GH), Auxiliary activities (AAs), Carbohydrate binding modules (CBM), Carbohydrate esterase (CE) and Polysaccharide K Lyase (PL). Only the top ten GT and GH families are shown.

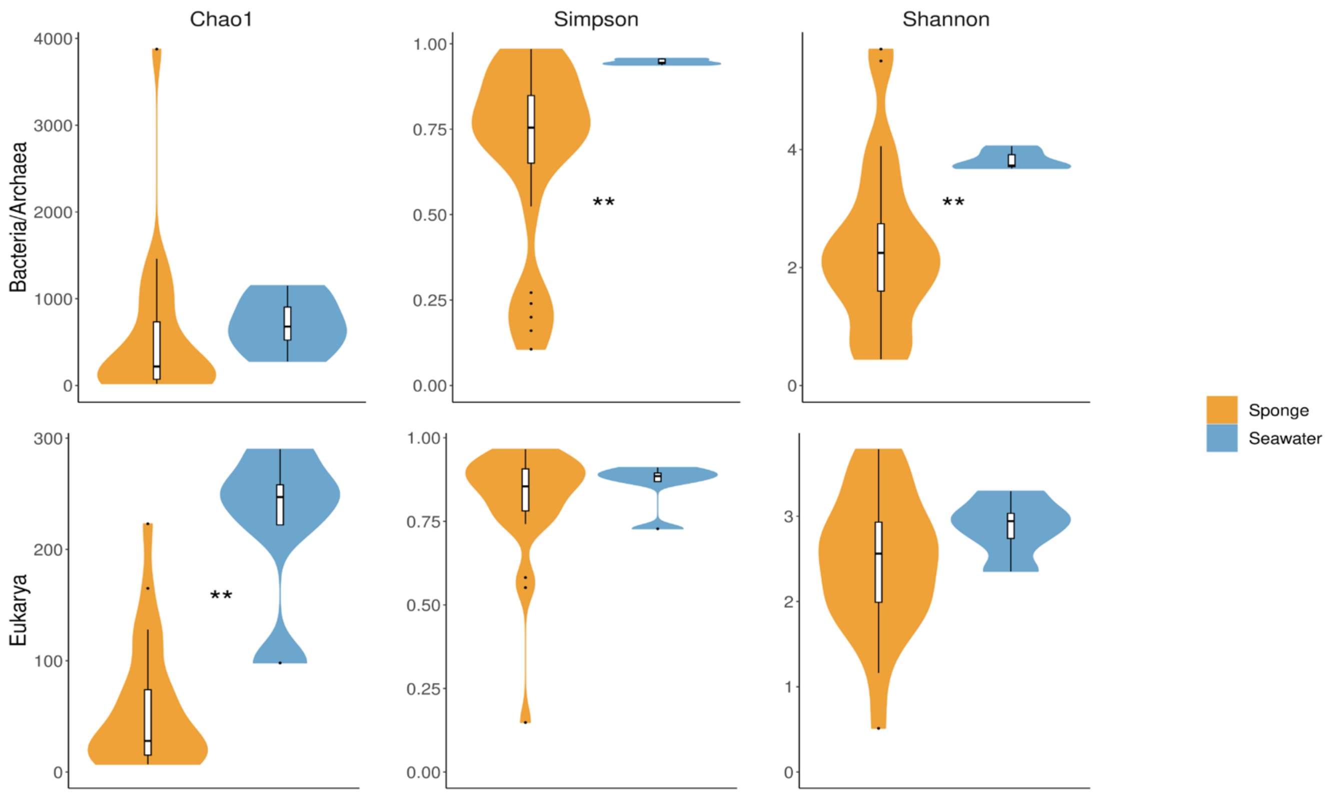

Supplementary Figure S1.

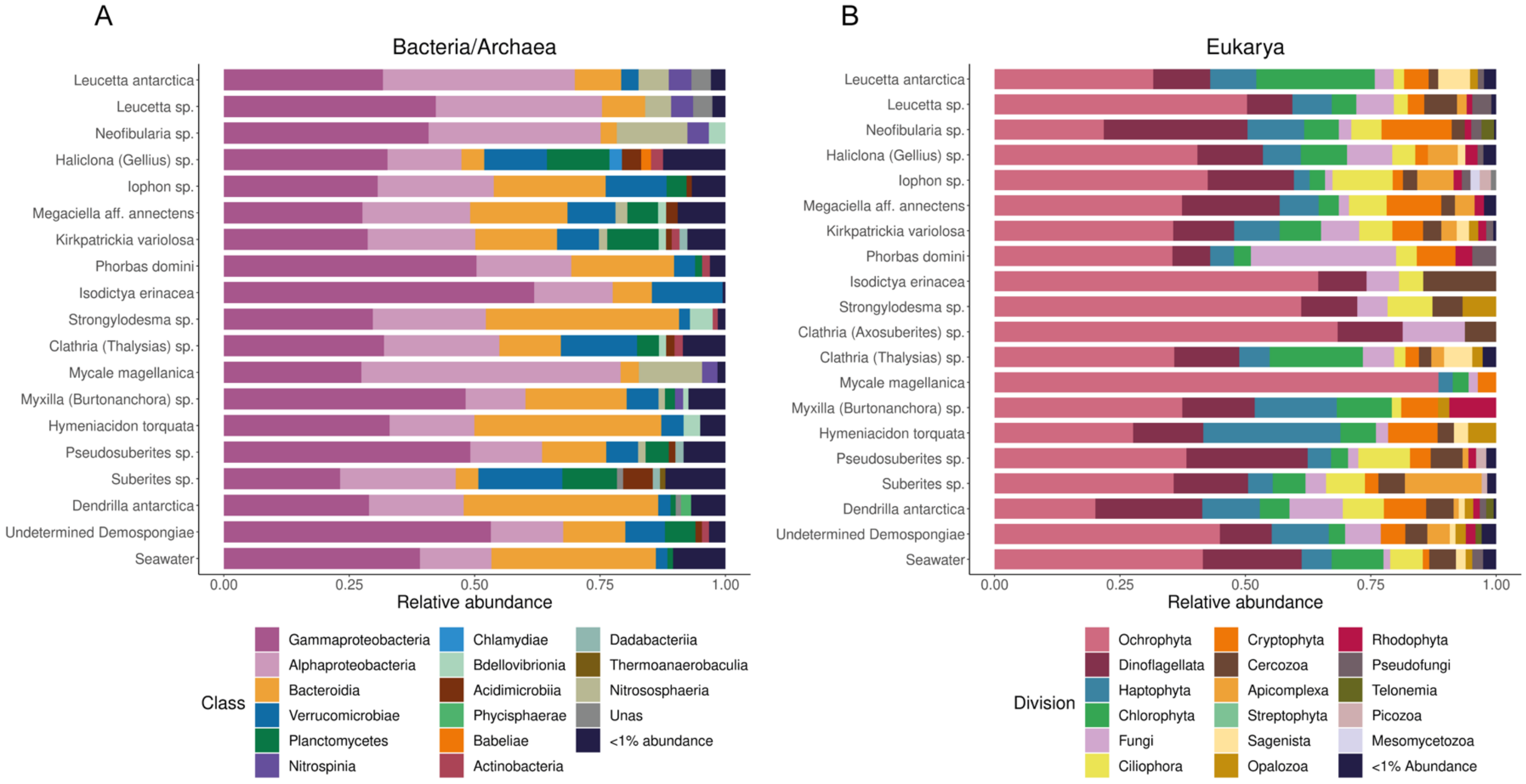

Supplementary Figure S2.

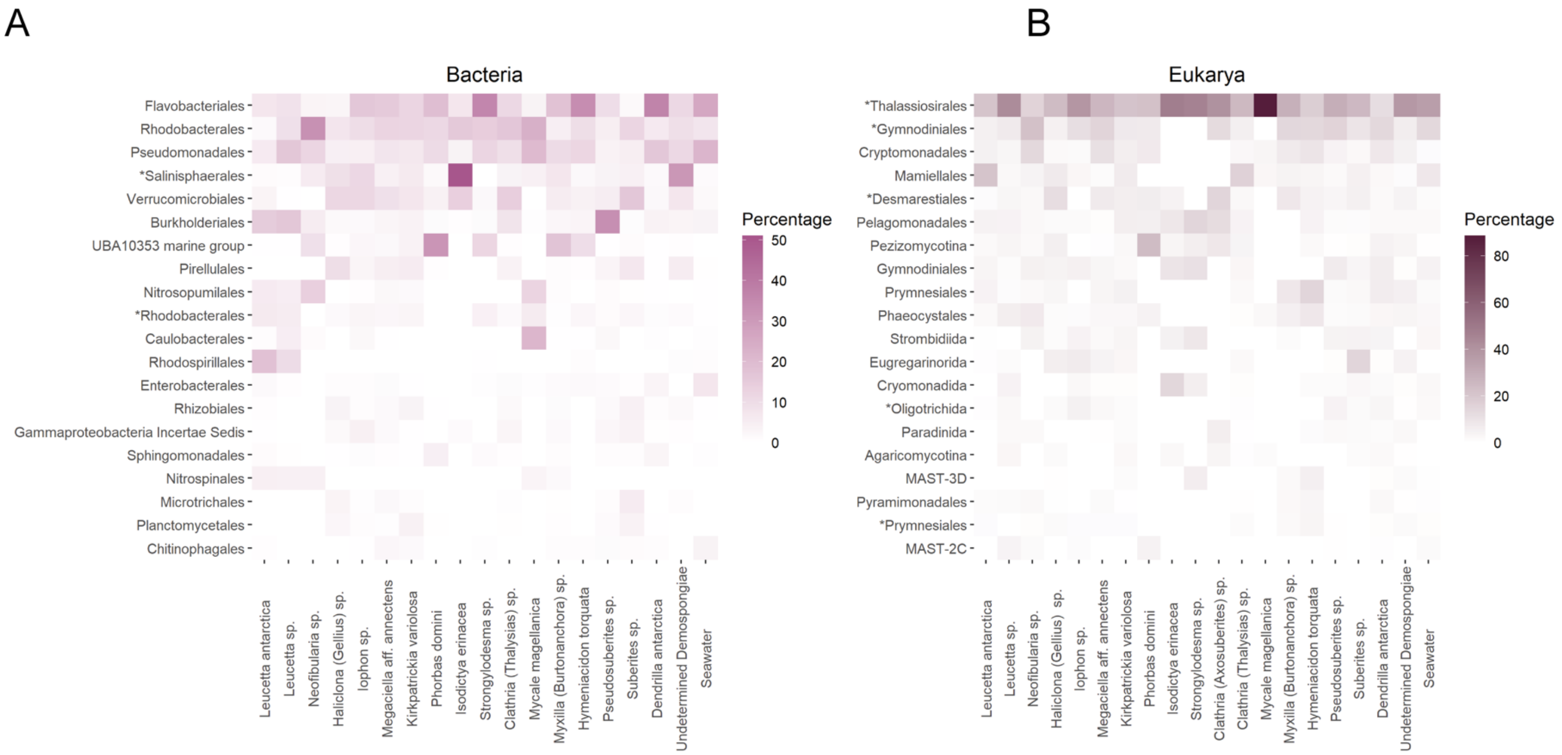

Supplementary Figure S3.

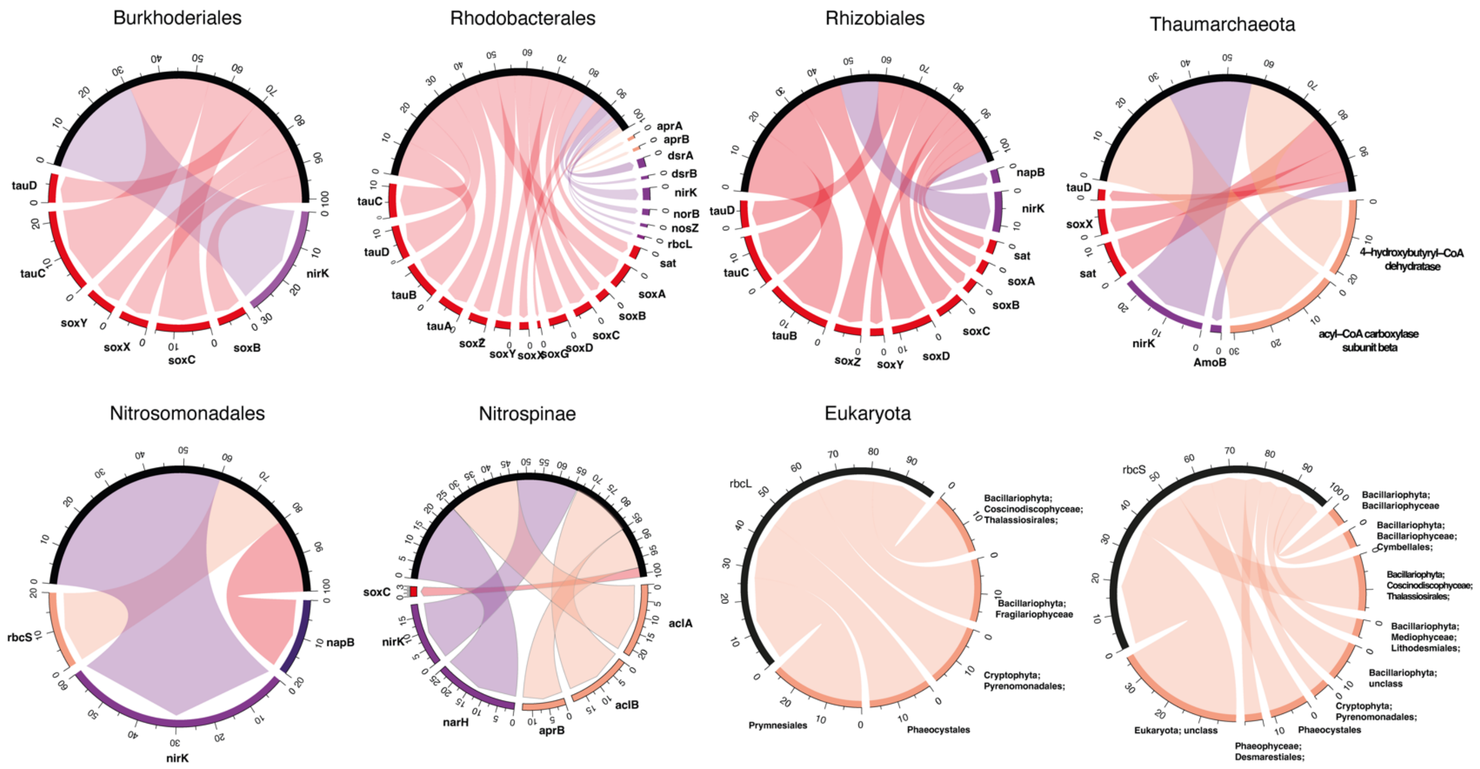

Supplementary Figure S4.

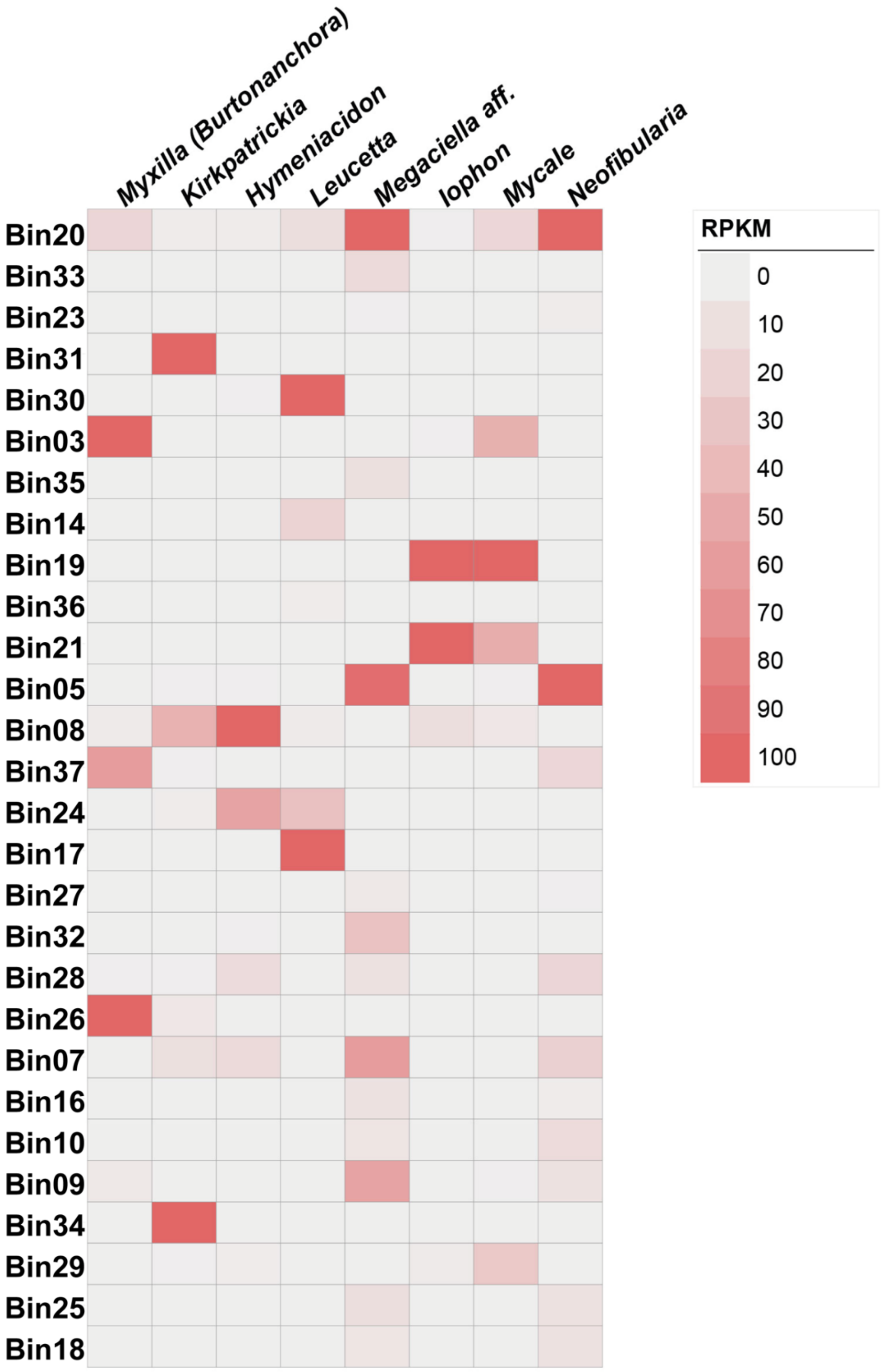

Supplementary Figure S5.

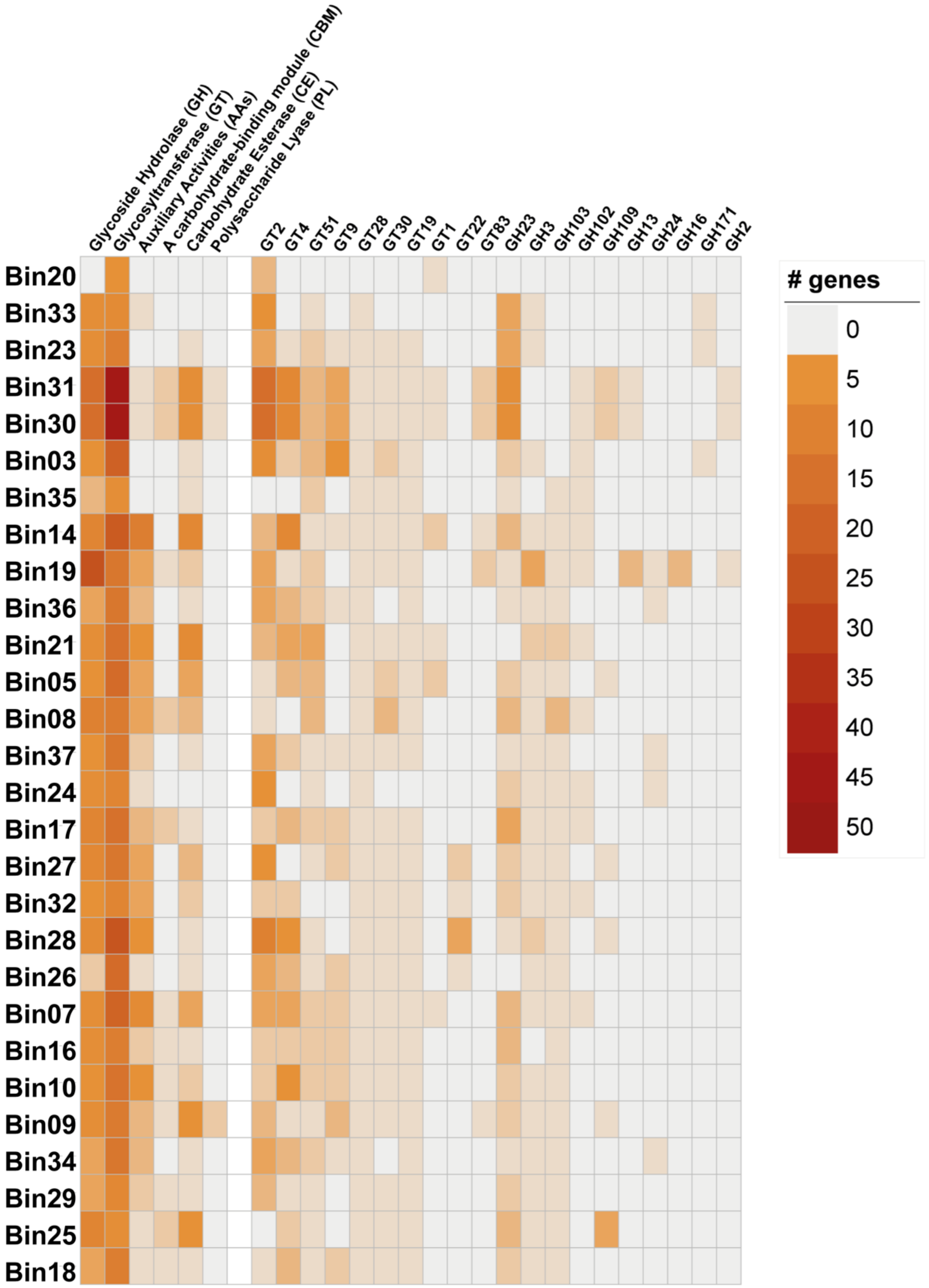

Supplementary Figure S6.

Supplementary Tables

Supplementary Table S1. Sponge and SW samples used in this study, indicating the samples that were used for tag sequencing (16S/18S rRNA) and metagenomics. The sample code, sampling date, depth, location, and detailed taxonomy of the sponge species analyzed are included.

Supplementary Table S2. Summary of tag sequencing analyses for 18S and 16S rRNA sequences and ecological indices (Chao1, Simpson, Shannon) for sponge and SW samples.

Supplementary Table S3. Topological properties of the co-occurrence networks of microbial communities associated with Antarctic sponges.

Supplementary Table S4. Summary of the metagenomic reads, assembly results and annotated sequences of the Antarctic sponge microbiomes

Supplementary Table S5. Taxonomic composition and relative abundance of microbial groups at genus-level based on metagenomic sequences.

Supplementary Table S6. Summary of functional genes annotated in general categories using eggNOG for the Antarctic sponge metagenomes.

Supplementary Table S7. Functional annotation and taxonomic classification at the protein level for the Antarctic sponge metagenomes.

Supplementary Table S8. Summary of the abundance of genes involved in vitamin production, symbiotic lifestyle, and cold adaptation genes analyzed in the sponge metagenomes. Gene abundance was computed using RPKG (reads per kilobase per genome equivalent) and normalized by average genome size.

Supplementary Table S9. Summary of taxonomic assignments of MAGs based on the GTDB database and their relative abundance computed in RPKM (reads per Kilobase per million mapped reads) based on recruitment analysis of the Antarctic sponge metagenomes.

Supplementary Table S10. Gene numbers involved in symbiosis, environmental adaptations, and numbers of carbohydrate-active enzymes (CAZy) detected in the genome of MAGs obtained from sponge metagenomes.
